# Resveratrol Protects Chondrocytes from Apoptosis via Altering the Ultrastructural and Biomechanical Properties: An AFM Study

**DOI:** 10.1371/journal.pone.0091611

**Published:** 2014-03-14

**Authors:** Hua Jin, Qian Liang, Tongsheng Chen, Xiaoping Wang

**Affiliations:** 1 Department of Pain Management, The First Affiliated Hospital of Jinan University, Guangzhou, China; 2 MOE Key Laboratory of Laser Life Science & Institute of Laser Life Science, South China Normal University, Guangzhou, China; LAAS-CNRS, France

## Abstract

Osteoarthritis (OA), a degenerative joint disease with high prevalence among older people, occurs from molecular or nanometer level and extends gradually to higher degrees of the ultrastructure of cartilage, finally resulting in irreversible structural and functional damages. This report aims to use atomic force microscopy (AFM) to investigate the protective effects of resveratrol (RV), a drug with good anti-inflammatory properties, on cellular morphology, membrane architecture, cytoskeleton, cell surface adhesion and stiffness at nanometer level in sodium nitroprusside (SNP)-induced apoptotic chondrocytes, a typical cellular OA model. CCK-8 assay showed that 100 μM RV significantly prevented SNP-induced cytotoxicity. AFM imaging and quantitative analysis showed that SNP potently induced chondrocytes changes including shrunk, round, lamellipodia contraction and decrease in adherent junctions among cells, as well as the destruction of biomechanics: 90% decrease in elasticity and 30% decrease in adhesion. In addition, confocal imaging analysis showed that SNP induced aggregation of the cytoskeleton and decrease in the expression of cytoskeletal proteins. More importantly, these SNP-induced damages to chondrocytes could be potently prevented by RV pretreatment. Interestingly, the biomechanical changes occurred before morphological changes could be clearly observed during SNP-induced apoptosis, indicating that the biomechanics of cellular membrane may be a more robust indicator of cell function. Collectively, our data demonstrate that RV prevents SNP-induced apoptosis of chondrocytes by regulating actin organization, and that AFM-based technology can be developed into a powerful and sensitive method to study the interaction mechanisms between chondrocytes and drugs.

## Introduction

Osteoarthritis (OA) is known as a degenerative arthritis or degenerative joint disease, which affects 20 million people in U.S. [1]. At present, the treatment for OA mainly focuses on relieving pains and symptoms, and improving function of cartilage. However, there are no treatments to cure OA or reduce the degradation of cartilage. Current treatments for OA are restricted to anti-inflammatory drugs which bring numerous side effects and are only temporarily effective to the patients. To find safe and highly effective drugs for OA treatment are therefore very urgent.

Resveratrol (3,5,4′ -trihydroxystilbene, RV), a polyphenol derived from grapes, berries, peanuts and other plants, has been shown to possess anti-proliferative, anti-oxidative and anti-inflammatory properties [2], and these effects are associated with the suppression of inflammation, arthritis and cardiovascular diseases [3]. It is reported that RV protects chondrocytes from apoptosis via preventing mitochondrial depolarization and ATP consumption [4] or suppressing ROS and p53-production [5]. RV also can be as a potent safe drug for OA treatment, but the mechanisms are still unclear.

Apoptosis of chondrocytes is regarded as a feature of progressive cartilage degeneration in OA [6]. Sodium nitroprusside (SNP) was widely used as the donor of nitric oxide (NO) to study the molecular mechanism of NO-induced chondrocytes apoptosis [7], [8]. Although NOC-12 may be a more effective NO donor in OA metastasis [9], it could not effectively induce apoptosis of chondrocytes [10]. Eo and co-workers [11] reported that RV could rescue SNP-induced degradation of I-kappa B alpha mainly through SN50 peptide-mediated inhibition of NF-kappa B activity, thus blocking SNP-induced caspase-3 activation and apoptosis. However, the effects of RV on morphological and biomechanical properties of chondrocytes at subcelluar or nanometer-level have not been studied.

Nanobiomechanics of cells have been identified as a vital characteristic to distinguish normal cells from diseased cells which differ physically from healthy cells [12]. Diseases can not only cause biological and functional alterations but also induce abnormalities in physical and structural characteristics of cells. Therefore, research into biomechanics at the cellular and molecular levels of some human diseases can provide a better elucidation on the mechanisms behind disease progression [13], thereby providing important information for treatment of these diseases as well. Due to the nanometer resolution, AFM has been extensively used in detection of some diseases at cellular or subcellular level [14]. The ultrastructural and biomechanical properties have been altered a lot in disease or cancerous cells, and these alterations can be used as target to diagnose or distinguish diseased cells from healthy cells [15], [16]. Furthermore, biomechanics of cell membrane is always changed in the context of drugs. Therefore, detecting these changes at nanometer level is very important for evaluating curative effect and elucidating mechanisms of drugs.

In this work, we used the rabbit chondrocytes as the cell model to detect the protective effects of RV on SNP-induced chondrocytes apoptosis. Rabbit chondrocytes have been extensively used in the basic research of mechanisms of chondrocytes or OA [17]–[24], and we have gained ripe experimental experiences [25]. Alterations in ultrastructure and biomechanics of cellular membrane of chondrocytes with or without RV pretreatment were investigated using AFM at nanometer scale. Our results showed that RV could effectively protect chondrocytes from apoptosis through altering the cytoskeleton arrangements and biomechanical properties including cellular stiffness and adhesion force.

## Materials and Methods

### Materials

Trypsin and type II collagenase, DMEM, fetal bovine serum, Cell Counting Kit-8 were purchased from Invitrogen (California, USA), Hyclone (Logan, Utah, USA), Sijiqing (Hangzhou, China) and Dojindo (Kumamoto, Japan), respectively. Actin-Tracker Green (phalloidin-FITC) and Tubulin-Tracker Red (α-Tubulin- Alexa Fluor 555) were both obtained from Beyotime Institute of Biotechnology (Naijing, China). Dulbecco's modified Eagle medium (DMEM) was from Gibco (Carlsbad, California, USA), fetal bovine serum (FBS) was from Sijiqing (Hangzhou, China).

### Isolation and culture of chondrocytes

New Zealand rabbits were purchased from Experimental Animal Center of Guangzhou (China). As Tonomura, et al [26] described, articular cartilage was derived from knee, hip and shoulder joints of 6-week-old New Zealand white rabbits. The utilization of rabbit articular cartilage has been approved by the Animal Ethics Committee of Guangdong province, China. The extracted cartilages were firstly minced into small pieces and Chondrocytes were isolated by enzymatic digestion of 0.25% Trypsin in phosphate buffered solution (PBS) for 1 h and 0.2% type II collagenase in DMEM for 4–6 h. After collection by centrifugation, chondrocytes were resuspended in DMEM supplemented with 10% FBS and antibiotics (100 U/ml penicillin and 100 U/ml streptomycin) and 4.5% glucose. The cells were transferred when confluent monolayer cells reached to 85–90%, the transferred density was 5×10^4^ cells/cm^2^. The growth medium was changed every other day. The second and third generations of chondrocytes were used in our study.

### CCK-8 assay to analyze cell viability

Chondrocytes were cultured in 96-well plates for 24 h, and then exposed to different concentrations of SNP for different periods. Cell viability was assessed using Cell Counting Kit assay according to the manufacturer's instructions. All experiments were performed three times.

### AFM measurements of cell morphology

For all topographic images, the cells were fixed with 2.5% paraformaldehyde, and imaged by a tapping mode AFM (Park Scientific Instruments) in air. The silicon nitride tips (UL20B) used in all AFM measurements were irradiated with ultraviolet in air for 15 min to remove any organic contaminates prior to use. The curvature radius of the tips is less than 10 nm, and the length, width and thickness of the cantilevers are 115, 30, and 3.5 μm, respectively, with the oscillation frequency of 255 kHz and a force constant of 0.03 N/m.

### Surface roughness of cell membrane

The average surface roughness (Ra) is defined as the arithmetic mean of the deviations in height from the line mean value, and Rq is the root mean square. As the roughness has a dependence on the sampling size, Ra and Rq were analyzed in two different areas: 10 randomly selected 4 μm^2^ (2 μm×2 μm) and 10 randomly selected 25 μm^2^ (5 μm×5 μm). P<0.05 were considered as statistical significance.

### Determination of nanomechanical properties of chondrocytes

The force spectroscopy of cells was detected using an AFM (Agilent 5500) in the near physiological environment. The methods here were according to Kim's procedure [27]. In brief, the cells were firstly fixed with 2.5% glutaraldehyde and kept in PBS (PH = 7.4) during AFM tip indentation. All force measurements were performed at the same loading rate (1.2×10^5^ pN/s). The deflection-vs-displacement curves were obtained by the instrument, and to convert the deflection-vs-displacement curves into the force-vs-distance curves, we adopted Cappella's method [28]. In each group, over 20 cells were measured. The data of stiffness and adhesion forces were processed using SPSS13.0 to gain the Gaussian distribution (or normal distribution) histograms.

The Young's modulus was calculated using Hertz model which shows the relationship between the applied force *F* and the indentation δ:
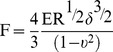



In the equation, *ν* is the poisson ratio, F the loading force, *δ* the indentation, *E* the Young's modulus, and *R* the curvature radius of the AFM tip, respectively. A Poisson ratio of 0.5 is appropriate for cells [29]–[31]. Young's modulus during the calculations to obtain the best fit to the model considering the least-squares method as proposed by Dimitriadis et al [32].

### Immunofluorescence staining

The characterizations of cytoskeleton were evaluated by staining with phalloidin-FITC and Tubulin-Tracker, separately. The chondrocytes were fixed with 4% paraformaldehyde for 30 min and incubated with 1 μM phalloidin-FITC or 1 μM Tubulin-Tracker for 60 min in dark at room temperature, separately, and then washed twice with PBS. After that, the cytoskeleton organization was imaged by a laser scanning confocal microscope (LCM 510 Meta Duo Scan, Carl Zeiss, Germeny). The resulting fluorescence was also measured by flow cytometer at excitation wavelength 488 nm, emission wavelength 530 nm to quantitatively elucidate the alterations of cytoskeleton proteins.

## Results and Discussions

### The changes in cell viability induced by SNP and RV

To detect the protecting effects of RV on chondrocytes, we firstly established the OA model by exposure of chondrocytes to SNP, an inorganic compound with the formula Na_2_[Fe(CN)_5_NO]•2H_2_O. SNP has been used as anti-hypertensive treatments for decades and it has not obvious side effects. In vitro, SNP could be as an external NO donor to induce apoptosis.

**Figure 1:**
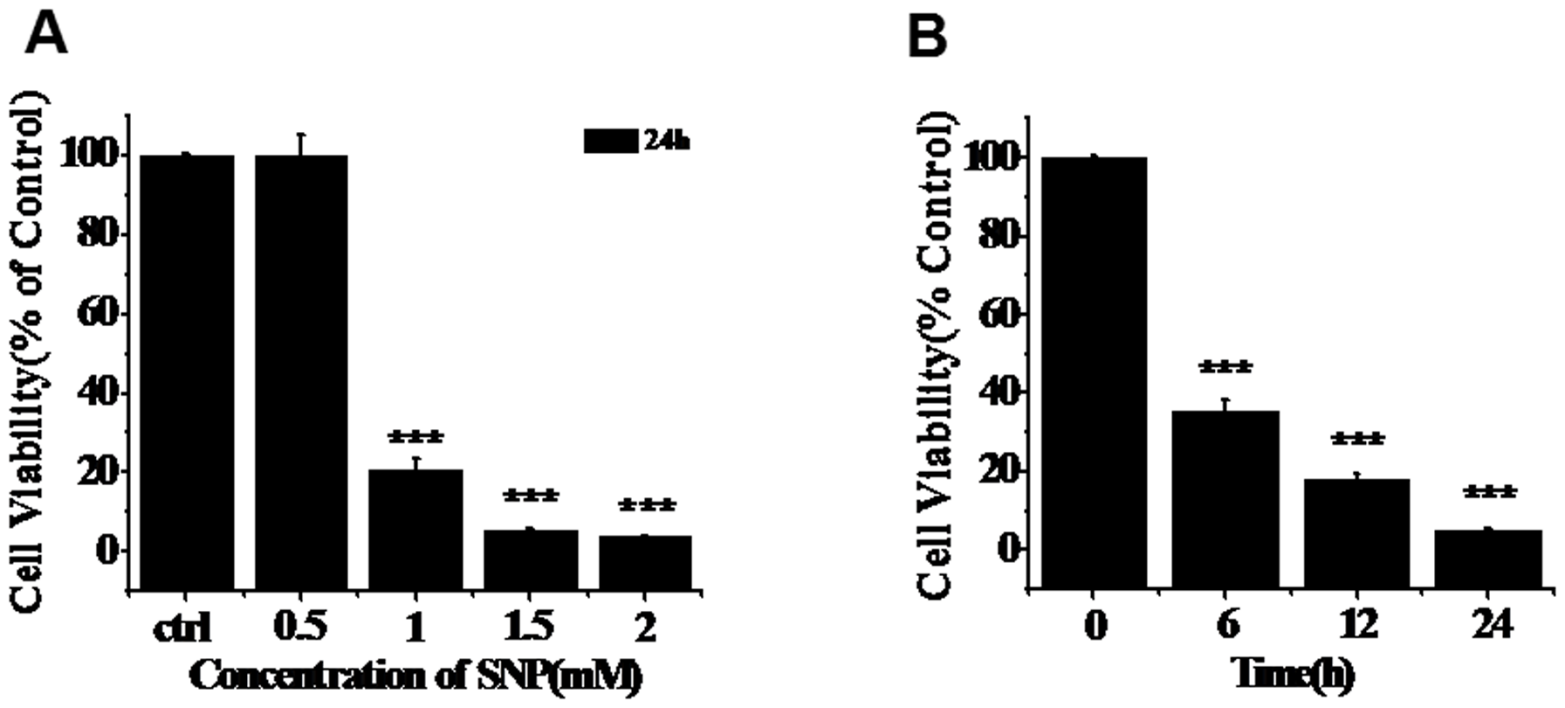
Cytotoxicity of SNP in chondrocytes. (A) *C*ell viability of chondrocytes treated by different concentrations of SNP for 24 h. (B) *C*ell viability of chondrocytes treated by 1.5 mM of SNP for different time periods (comparing with control group, *P<0.05, **P<0.01, ***P<0.001). The results indicated the killing effects of SNP on chondrocytes were in a dose- and time-dependent manner.

As shown in Figure 1A, SNP induced a dose-dependent cytotoxicity in chondrocytes, and treatment with 1.5 mM SNP for 24 h induced an over 85% of decrease of cell viability. Treatment with 1.5 mM of SNP for different time induced a time-dependent cytotoxicity (Fig.1B). The results indicated that SNP induced dose- and time-dependent cytotoxicity in chondrocytes. Based on these data, chondrocytes treated with 1.5 mM SNP for 24 h were used as the in vitro OA model.

**Figure 2:**
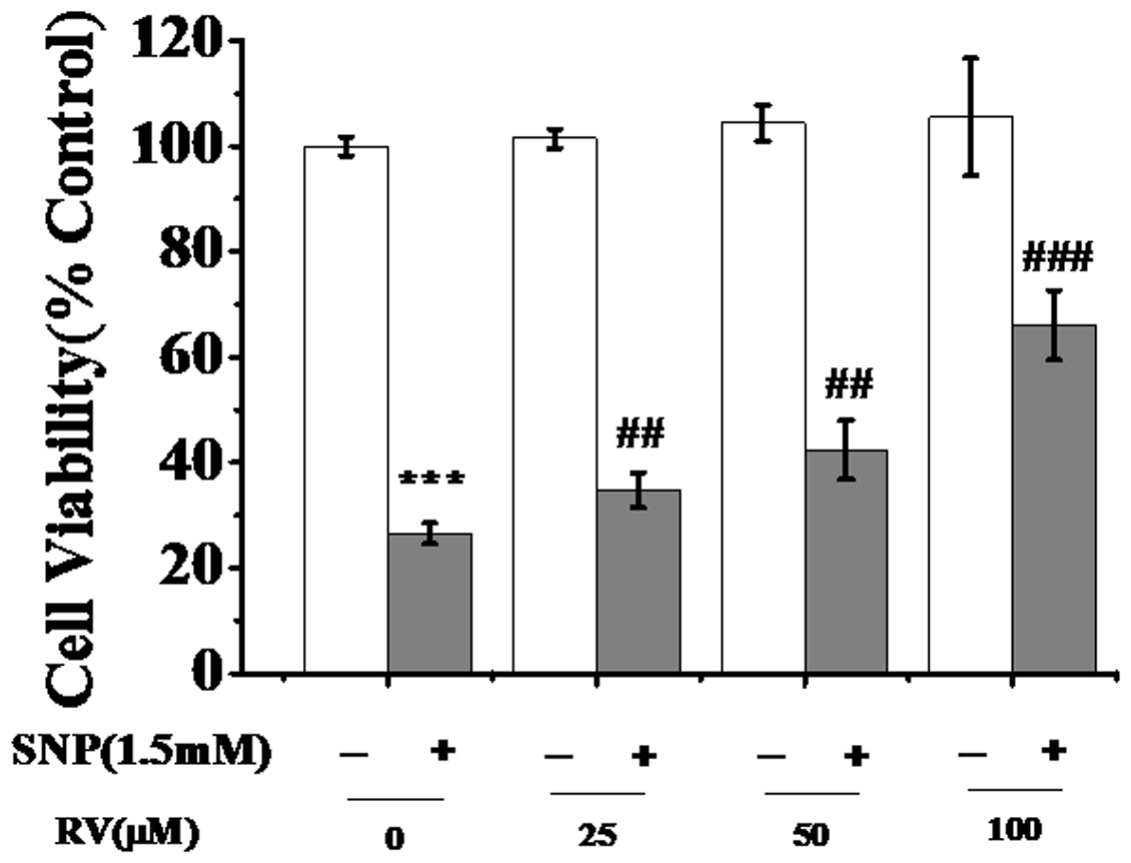
Protection effects of RV on SNP-induced apoptosis of chondrocytes. Cells were pretreated with different concentrations (0, 25, 50 and 100 mM) of RV for 24 h, and then treated with 1.5 mM of SNP for 12 h. After that, the cell viability was assayed using CCK-8 (comparing with control group, *P<0.05, **P<0.01; comparing with SNP treated group, #P<0.05, ##P<0.01, ###P<0.001).

To investigate the effects of RV on chondrocytes, different concentrations of RV were used to pretreat chondrocytes for 24 h before SNP treatment, and then the cell viability was measured. As shown in Figure 2, RV not only did not significantly induce cytotoxicity but also potently prevented SNP-induced cytotoxicity (Fig. 2), indicating that RV could protect chondrocytes from SNP-induced apoptosis and could be used as a potent drug to treat OA.

### AFM detects morphological changes of chondrocytes

The specific shape of cells plays vital roles in maintaining specific functions of cells. Cellular shapes and morphology determine the interaction extent between cells and their environment. If cellular shapes were changed, the physiological and functional situations of cells would be damaged or disturbed [33], [34]. Therefore, detecting the structural details of chondrocytes is very helpful for understanding their functions. Morphological data of cells obtained using AFM could be an important index to evaluate the effects of drugs [35].

**Figure 3:**
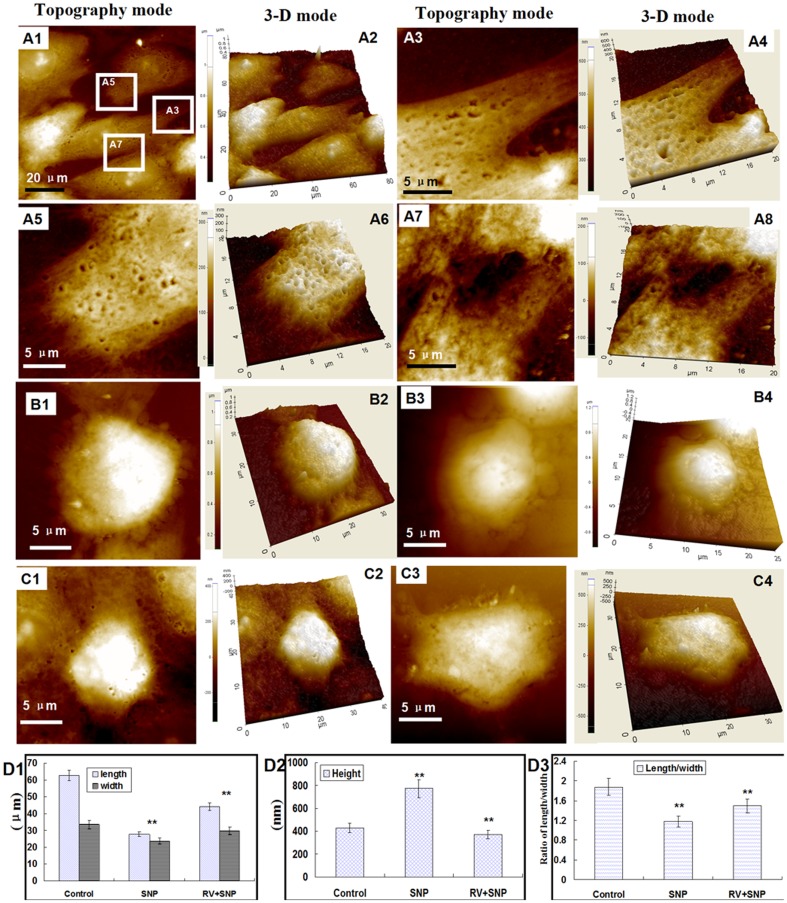
Morphological data of chondrocytes. (A1–A2) Control chondrocytes. (A3–A8) The enlargement images of white panes in A1. (B1–B4) Chondrocytes treated with 1.5 mM SNP for 12 h. (C1–C5) The chondrocytes were pretreated with 100 mM of RV for 24 h, and then treated with 1.5 mM of SNP for 12 h. (D1–D3) Histograms of average length, width (D1), height (D2) and ratio of length/width of cells in three groups. In D1–D3, more than ten cells in each group were selected to measure the values. *P<0.05 was regarded as statistically significant.

As shown in Figure 3, control chondrocytes were spindle and elongated shapes (A1, A2), and lots of lamellipodia and filopodia were observed around the cells (A3, A4, A5, A6). In addition, the adherent junctions between/among cells were connected by lamellipodia (A7, A8), which provided essential condition for the exchange of energy and matter among chondrocytes. After treatment with 1.5 mM of SNP for 12 h, chondrocytes become shrunk and round, the lamellipodia contracted, and the adherent junctions among cells significantly decreased or even diminished (shown by B1–B4), the characterizations of apoptosis. These SNP-induced changes were potently prevented by pretreatment with 100 μM of RV for 24 h (shown by images C1–C4), demonstrating that RV pretreatment could markedly prevent SNP-induced morphological changes and apoptosis of chondrocytes.

Besides, quantitative morphological data were also compared for accurate assessment of the effects of RV. As shown in Figure 3D1, the average length and width of control chondrocytes were 62.8±5.2, 33.5±2.6 μm, and they decreased to 27.8±2.1 and 23.6±3.3 μm after treatment with SNP. In addition, SNP treatment induced a significant increase in the average height of chondrocytes from 427.4±44.6 nm (control) to 774.8±85.6 nm (Fig. 3D2), and also induced a significant decrease in the ratio of major and minor cell axis from 1.9±0.2 (control) to 1.2±0.1 (Fig.3D2). Both the decreased width and increased height indicated that the cells were tending to detach from the cellular matrix and became shrank round, even apoptosis. More importantly, all these morphological changes induced by SNP were potently prevented by RV pretreatment (shown by Fig. 3D1, D2).

Taken together, RV pretreatment could significantly prevented SNP-induced apoptosis of chondrocytes by protecting cellular structure, shape and biomechanics.

### Alterations in cellular membrane architecture detected at nanometer level

**Figure 4:**
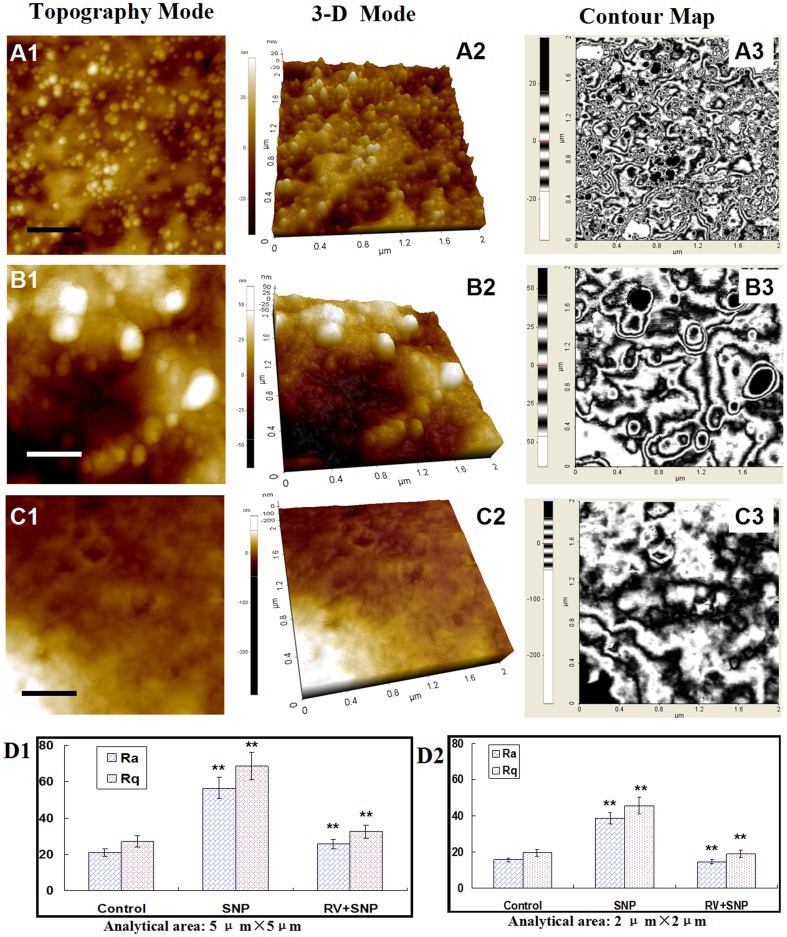
AFM ultrastructural data of control chondrocytes. (A1–A3) Control chondrocytes. (B1–B3) Chondrocytes treated with 1.5 mM SNP for 12 h. (C1–C3) The chondrocytes were pretreated with 100 μM of RV for 24 h, and then treated with 1.5 mM of SNP for 12 h. Scanning area: 2×2 μm^2^. (A1), (B1), (C1) was topography mode. (A2), (B2), (C2) 3-D mode of (A1), (B1) and (C1), respectively. (A3), (B3), (C3) was contour map of (A1), (B1) and (C1), respectively. (D1) and (D2) were histograms of average roughness (Ra) of chondrocytes which were analyzed in 5×5 μm^2^ and 2×2 μm^2^, respectively. In (D1) and (D2), ten cells in each group were selected to measure the values of Ra, statistical analysis was performed using Student's *t*-test. P<0.05 was regarded as statistically significant.

Figure 4 showed the ultrastructural data of chondrocytes. The membrane architecture of control chondrocytes (Figs.4A1–A3) showed uniform structures and granular morphology with the surface particles of 50∼100 nm. Figures 4B1–B3 showed the surface architecture of SNP-induced chondrocytes which became heterogeneous, and the sizes of the membrane particles increased to 150∼200 nm. The ultrastructure of SNP-treated chondrocytes pretreated by RV became smooth and homogeneous but the granular morphology on cellular membrane diminished (Figs.4C1–C3), implying that RV could significantly protect chondrocytes from SNP-induced apoptosis and changes in morphological properties, but could not completely prevented SNP-induced changes in nanostructure of cellular membrane. Therefore, AFM with nanometer-scale resolution provides us new insights about cellular structure-function.

Additionally, the average roughness of cell membrane is directly or indirectly sensitive to the membrane-skeleton integrity [36]. The average roughness (Ra) and root mean square roughness (Rq) were measured for comparison. As shown in Figures 4D1 and D2, the values of both Ra and Rq of SNP-induced chondrocytes increased significantly compared with that of control chondrocytes. While Ra and Rq of the -chondrocytes pretreated with RV decreased 50% than that of SNP group, and their values were similar to that of control chondrocytes. These data suggested that RV protected chondrocytes from SNP-induced damages via altering the membrane architecture.

Taken together, all these morphological data revealed that SNP could successfully induced apoptosis in chondrocytes, and RV could protect the chondrocytes from damaging or apoptosis via changing their morphological properties and architectures.

### Alterations in nanomechanical properties of chondrocytes

Since the biomechanical properties of cells can potentially indicate their function and health, it is therefore very important to study the cellular biomechanics. Lots of literatures have shown that study on cellular mechanics is very helpful for clinical diagnostics and even the formulation of suitable strategies towards effective therapeutic treatments of human diseases. Although AFM measures the nanobiomechanics of single cell, it has been used to diagnose some diseases [16], [37]. Although the potent protection of RV on chondrocytes has been reported [38], [39], the effects on the nanobiomechanics, particularly on cell function and growth, is poor.

**Figure 5:**
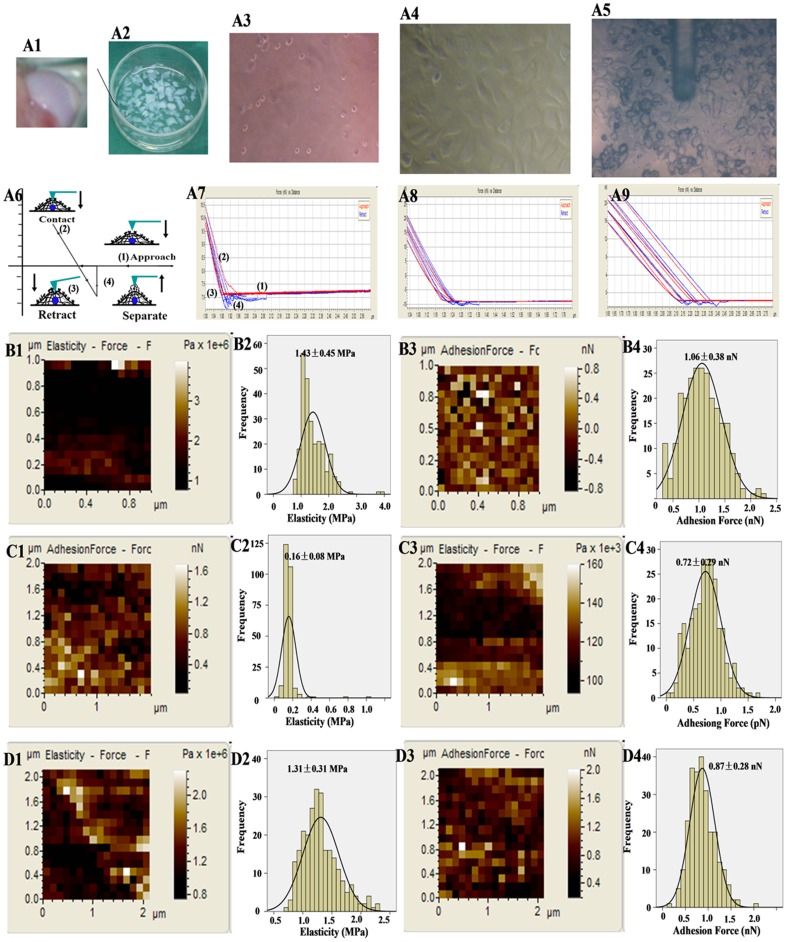
Alterations in nanobiotechnology of chondrocytes detected by AFM. (A1–A5) isolation of chondrocytes: (A1) Cartilage collected from the bilateral joints of the knees, hips, and shoulders. (A2) The joints were minced into small pieces, treated with 0.015% trypsin for 30 min, and subsequently digested. (A3) Morphology of primary joint chondrocytes. (A4) The morphology of primary joint chondrocytes cultured for 7 days. (A5) The AFM tip was employed to detect the morphology and biomechanics of chondrocytes. (A6) Typical force-distance curve detected using AFM: (1) The tip is approaching the surface of sample, (2) the tip is just in contact with the surface of cells, (3) the tip is further put into repulsive contact with the cellular surface, (4) lastly, the tip-sample contact is retracted. (A7–A9) are the representative force-distance curves obtained on control chondrocytes (A7), chondrocytes treated with 1.5 mM SNP for 12 h (A8), and chondrocytes pretreated with RV and the induce with SNP (A9), respectively. The elasticity maps, histogram of elasticity, adhesion force map and histogram of adhesion force of control chondrocytes (B1–B4), chondrocytes treated with 1.5 mM SNP for 12 h (C1–C4), and chondrocytes pretreated with RV and then cotreated with SNP (A4), respectively.

Here, the nanobiomechanical properties including elasticity and adhesion force were detected at levels of nanometer and pN, respectively. Figures 5A1–A4 showed the isolation of chondrocytes. Figures A5 and A6 indicated that the AFM tip was employed to detect the morphology and biomechanics of chondrocytes. After positioning the AFM tip over the cell center (Fig.5A5), the tip was brought to contact and pressed against the cell surface (Fig.5A6). During the tip retraction from the cell surface rupture events, the retraction events in the force-distance curves revealed the general tip-cell-surface adhesive interactions. Figs 5A7–A9 showed the typical force-distance curves obtained from control chondrocytes, SNP and RV+SNP groups, respectively.

As shown in Figs 5B1 and B2, the elasticity/stiffness of control chondrocytes was 1.43±0.45 MPa. After treatment with 1.5 mM SNP for 12 h, the elasticity of the chondrocytes decreased to 0.16±0.08 MPa (Figs.5C1 andC2), indicating that SNP obviously destructed the rigidity and chemical compositions of chondrocytes membrane. Nevertheless, if the chondrocytes pretreated with 100 μM of RV for 24 h before exposure to 1.5 mM SNP for 12 h, the elasticity was 1.31±0.31 MPa (Fig.5D1,D2), indicating that RV could effectively protect the elasticity/stiffness of chondrocytes. The damage to the cell envelope and the changes in the composition of chondrocytes induced by SNP were suspected to be the major causes of the decreases in the cell stiffness/elasticity. As reported by Cai and co-workers [40], the damage and destruction of the cytoskeleton directly led to the decrease of cellular rigidity.

Moreover, adhesion of cellular membrane plays a very important role in cell physiological and pathological processes [41]. Here, we employed force spectroscopy of AFM to measure the non-specific adhesion force between AFM tip and cellular membrane as a function of the nanomechanical properties of the existing surface adhesive molecules. The adhesion force of control chondrocytes was 1.06±0.38 pN (Figs.5B3 and B4), but it decreased to 0.72±0.29 pN after SNP treatment (Figs.5C3 and C4), indicating that membrane proteins were damaged by SNP treatment. However, the adhesion force of SNP-treated chondrocytes pretreated with RV only increased to 0.87 ±0.28 pN, demonstrating that RV could partially protect the membrane proteins. Furthermore, RV significantly increased the number of actins (A1–A4), and the particles with nano-meter scale were mainly distributed on/around actins (B1–B3) (Fig.S1). Notably, the nanomechanical properties of cellular membrane, including adhesion force and stiffness, were both enhanced with RV treatment (Figs.S1 C1 and C2).

Taken together, these results showed that SNP induced the destruction of biomechanics in chondrocytes, including 90% decrease in elasticity and 30% decrease in adhesion. Notably, RV pretreatment could recover the elasticity/stiffness closely to that of control chondrocytes, but RV possessed only a little protecting effect on membrane proteins, implying that RV could not entirely protect the physiological and functional properties of chondrocytes.

### Alterations in cytoskeletal proteins F-actin and α-tubulin

From the biomechanical data, we can see that RV protects chondrocytes from SNP-induced apoptosis mainly through maintaining their elasticity/stiffness. As cytoskeleton is very important to maintain the biomechanics of cells, we further qualitatively investigated the organization of cytoskeleton, including F-actin and α-tubulin using confocal microscopy. The cytoskeleton is the mesh-like structure beneath the cell membrane, which is an important reflection of cellular structure organization [42]. Particularly, F-actin cytoskeleton is extensively regarded as the key factor to regulate the shapes and generate the mechanical forces of cells, and it plays vital roles during cellular physiological and pathological behaviors.

**Figure 6:**
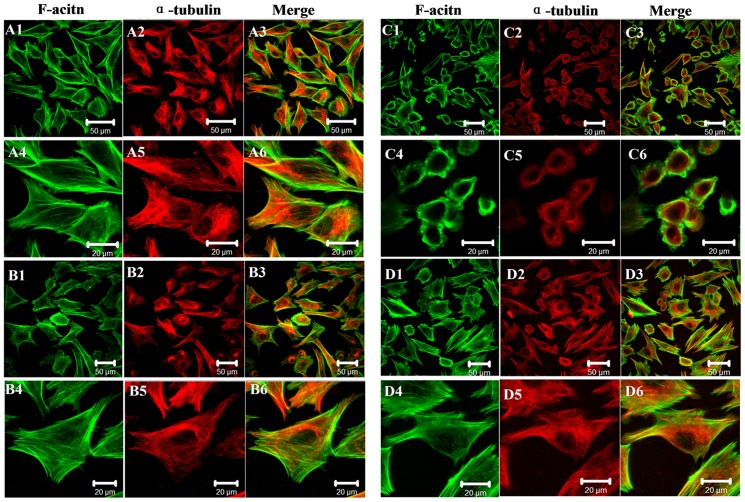
Organization of cytoskeleton chondrocytes. Control chondrocytes (A1–A6), chondrocytes treated with 100 μM RV for 12 h (B1–B6), chondrocytes treated with 1.5 mM SNP for 24 h (C1–C6) and chondrocytes pretreated with RV and then treated with SNP (D1–D6), respectively. A4–A6, B4–B6, C4–C6, D4–D6 were enlarged images of cellular cytoskeleton in A1–A3, B1–B3, C1–C3, D1–D3, respectively. Bars in A1–A3, B1–B3, C1–C3, D1–D3 and A4–A6, B4–B6, C4–C6, D4–D6 were 50 and 20 μm, respectively.

As shown in Figure 6, control chondrocytes presented well-spreading shapes, and F-actin and α-Tubulin were well-organized uniformly assembly (Figs.6A1–A6). The F-actins were of paralleled-like organization and α-Tubulin microtubules were of mesh-like alignment, which showed a well-grown station of control chondrocytes. After exposure of cells to 100 μM RV for 24 h, the fluorescence intensity increased significantly (Figs.6B1–B6) indicative of the increase of both F-actins and α-Tubulin, suggesting that RV treatment may protect the cellular cytoskeleton and promote the expression of cytoskeletal proteins. While after treatment with SNP, the chondrocytes became shrunk and round, and both the F-actin and α-Tubulin cytoskeleton were reorganized and polymerized (Figs.6C1–C6 showed). Interestingly, in the RV-pretreated chondrocytes, we found that the cytoskeleton could recover to some extent and represented well-spreading organizations (shown by Figs.6D1–D6), indicating that RV protected chondrocytes from SNP-induced apoptosis mainly through altering the organization of cytoskeleton. For further detecting the expression of cytoskeletal proteins induced by SNP and RV, we also measured the MFI of F-actin and α-tubulin using fluorescence-based flow cytometry, and found that SNP induced apoptosis and stiffness decrease mainly through the reorganization and decrease the cytoskeletal proteins—F-actin instead of α-tubulin (Fig.S2).

All these data demonstrated that the biomechanical properties were heavily influenced by the organization and expression levels of actin microfilaments instead of α-tubulin. SNP induced aggregation of cytoskeleton, decrease in the expression of cytoskeletal proteins, 90% decrease of elasticity and 30% decrease of adhesion force in chondrocytes. While RV pretreatment could protect the elasticity/stiffness close to that of control chondrocytes through protecting the integrity and organization of cell cytoskeleton. These data demonstrate that AFM can be as a promising nanodevice to study cells cytoskeleton integrity and arrangement in vitro models of apoptosis and migration.

It is very imperative to evaluate the effects of RV on chondrocytes when it is used in tissue engineering or OA treatment. The evidence provided in this study showed that RV could protect chondrocytes from SNP-induced apoptosis by regulating actin organization, but it had only a little effect on adhesive molecules or proteins in/on cell membrane, and that AFM-based technique provides us an effective and feasible tool to detect the changes and underlying mechanism of cells induced by drugs at nanometer level.

## Supporting Information

Figure S1 The morphological and nanomechanical properties of chondrocytes treated with 100 nM of RV for 24 h, which detected using AFM. A1–A2 Morphology of chondrocytes. A3–A4 the enlargement of white frame in A1. B1–B3 The ultrastructure of membrane on chondrocytes. C1,C2 The adhesion force and stiffness modulus of cellular membrane was 2.13±0.66 nN and 5.05±2.43 MPa.
(TIF)

Figure S2 The alterations in expression of F-actin andα-tubulin in chondrocytes treated with SNP, RV and SNP+RV, respectively, which detected by fluorescence-based flow cytometry. The F-actins and microtubule were stained with FITC- phalloidine and Alexa Fluor 555-α-Tubulin antibody, respectively.
(TIF)

## References

[1] Anderson AS, Loeser RF (2010) Why is osteoarthritis an age-related disease? Best Pract Res Cl Rh 24(1): 15–26. 10.1016/j.berh.2009.08.006 PMC2818253 20129196

[2] Wadsworth TL, Koop DR (1999) Effects of the wine polyphenolics quercetin and resveratrol on pro-inflammatory cytokine expression in RAW 264.7 macrophages. Biochem Pharmacol 57(8): 941–949. 10086329 10.1016/s0006-2952(99)00002-7

[3] Shakibaei M, Harikumar KB, Aggarwal BB (2009) Reseratrol addiction: to die or not to die. Mol Nutr Food Res 53(1): 115–128. 19072742 10.1002/mnfr.200800148

[4] Dave M, Attur M, Palmer G, Al-Mussawir HE, Kennish L, et al (2008) The antioxidant resveratrol protects against chondrocyte apoptosis via effects on mitochondrial polarization and ATP production. Arthritis Rheum 58(9): 2786–2797. 18759268 10.1002/art.23799

[5] Csaki C, Keshishzadeh N, Fischer K, Shakibaei M (2008) Regulation of inflammation signalling by resveratrol in human chondrocytes in vitro. Biochem Pharmacol 75(3): 677–687. 17959154 10.1016/j.bcp.2007.09.014

[6] Del Carlo M Jr, Loeser RF (2008) Cell death in osteoarthritis. Curr Rheumatol Rep 10(1): 37–42. 18457610 10.1007/s11926-008-0007-8

[7] Wu GJ, Chen TG, Chang HC, Chiu WT, Chang CC, et al (2007) Nitric oxide from both exogenous and endogenous sources activates mitochondria-dependent events and induces insults to human chondrocytes. J Cell Biochem 101(6): 1520–1531. 17492650 10.1002/jcb.21268

[8] Nakagawa S, Arai Y, Mazda O, Kishida T, Takahashi KA, et al (2010) N-acetylcysteine prevents nitric oxide-induced chondrocyte apoptosis and cartilage degeneration in an experimental model of osteoarthritis. J Orthopaed Res 28(2): 156–163. 10.1002/jor.20976 19725096

[9] Andrés MC de, Maneiro E, Martín MA, Arenas J, Blanco FJ (2013) Nitric oxide compounds have different effects profiles on human articular chondrocyte metabolism. Arthritis Res Ther 15(5): R115. 24025112 10.1186/ar4295 PMC3978712

[10] Carlo MD, Loeser RF (2003) Increased oxidative stress with aging reduces chondrocyte survival: correlation with intracellular glutathione levels. Arthritis Rheum 48(12): 3419–3430. 14673993 10.1002/art.11338

[11] Eo SH, Cho H, Kim SJ (2013) Resveratrol Inhibits Nitric Oxide-Induced Apoptosis via the NF-Kappa B Pathway in Rabbit Articular Chondrocytes. Biomol Ther 21(5): 364–370. 10.4062/biomolther.2013.029 PMC3825200 24244824

[12] Lee GY, Lim CT (2007) Biomechanics approaches to studying human diseases. Trends Biotechnol 25(3): 111–118. 17257698 10.1016/j.tibtech.2007.01.005

[13] Stolz M, Gottardi R, Raiteri R, Miot S, Martin I, et al (2009) Early detection of aging cartilage and osteoarthritis in mice and patient samples using atomic force microscopy. Nat nanotechnol 4(3): 186–192. 19265849 10.1038/nnano.2008.410

[14] Stolz M, Raiteri R, Daniels AU, VanLandingham MR, Baschong W, et al (2004) Dynamic elastic modulus of porcine articular cartilage determined at two different levels of tissue organization by indentation-type atomic force microscopy. Biophys J 86(5): 3269–3283. 15111440 10.1016/S0006-3495(04)74375-1 PMC1304192

[15] Iyer S, Gaikwad RM, Subba-Rao V, Woodworth CD, Sokolov I (2009) Atomic force microscopy detects differences in the surface brush of normal and cancerous cells. Nat Nanotechnol 4(6): 389–393. 19498402 10.1038/nnano.2009.77 PMC3079421

[16] Cross SE, Jin YS, Tondre J, Wong R, Rao J, et al (2008) AFM-based analysis of human metastatic cancer cells. Nanotechnology 19: 384003–384011. 21832563 10.1088/0957-4484/19/38/384003

[17] Kim SJ, Ju JW, Oh CD, Yoon YM, Song WK, et al (2002) ERK-1/2 and p38 kinase oppositely regulate nitric oxide-induced apoptosis of chondrocytes in association with p53, caspase-3, and differentiation status. J Biol Chem 277(2): 1332–1339. 11689560 10.1074/jbc.M107231200

[18] Kim SJ, Kim HG, Oh CD, Hwang SG, Song WK, et al (2002) p38 kinase-dependent and -independent inhibition of protein kinase C ζ and -α regulates nitric oxide-induced apoptosis and dedifferentiation of articular chondrocytes. J Biol Chem 277(33): 30375–30381. 12048219 10.1074/jbc.M205193200

[19] Kim SJ, Hwang SG, Shin DY, Kang SS, Chun JS (2002) p38 Kinase Regulates Nitric Oxide-induced Apoptosis of Articular Chondrocytes by Accumulating p53 via NFkappaB-dependent Transcription and Stabilization by Serine 15 Phosphorylation. J Biol Chem 277(36): 33501–33508. 12091386 10.1074/jbc.M202862200

[20] Oh CD, Chun JS (2003) Signaling mechanisms leading to the regulation of differentiation and apoptosis of articular chondrocytes by insulin-like growth factor-1. J Biol Chem 278(38): 36563–36571. 12853454 10.1074/jbc.M304857200

[21] Kim SJ, Hwang SG, Kim IC, Chun JS (2003) Actin cytoskeletal architecture regulates nitric oxide-induced apoptosis, dedifferentiation, and cyclooxygenase-2 expression in articular chondrocytes via mitogen-activated protein kinase and protein kinase C pathways. J Biol Chem 278(43): 42448–42456. 12907684 10.1074/jbc.M304887200

[22] Yoon JB, Kim SJ, Hwang SG, Chang S, Kang SS, et al (2003) Non-steroidal anti-inflammatory drugs inhibit nitric oxide-induced apoptosis and dedifferentiation of articular chondrocytes independent of cyclooxygenase activity. J Biol Chem 278(17): 15319–15325. 12588866 10.1074/jbc.M212520200

[23] Hwang SG, Ryu JH, Kim IC, Jho EH, Jung HC, et al (2004) Wnt-7a causes loss of differentiated phenotype and inhibits apoptosis of articular chondrocytes via different mechanisms. J Biol Chem 279(25): 26597–26604. 15082716 10.1074/jbc.M401401200

[24] Kim JS, Park ZY, Yoo YJ, Yu SS, Chun JS (2005) p38 kinase mediates nitric oxide-induced apoptosis of chondrocytes through the inhibition of protein kinase C by blocking autophosphorylation. Cell Death Differ 12(3): 201–212. 15665819 10.1038/sj.cdd.4401511

[25] Zhuang CP, Wang XP, Chen TS (2013) H_2_O_2_ induces apoptosis of rabbit chondrocytes via both the extrinsic and the caspase-independent intrinsic pathways. J Innov Opt Health Sci 6(3): 1350022.

[26] Tonomura H, Takahashi KA, Mazda O, Arai Y, Inoue A, et al (2006) Glutamine protects articular chondrocytes from heat stress and NO-induced apoptosis with HSP70 expression. Osteoarthritis Cartilage 14(6): 545–553. 16480901 10.1016/j.joca.2005.12.008

[27] Kim KS, Cho CH, Park EK, Jung MH, Yoon KS, et al (2012) AFM-detected apoptotic changes in morphology and biophysical property caused by paclitaxel in Ishikawa and HeLa cells. PloS one 7(1): e30066. 22272274 10.1371/journal.pone.0030066 PMC3260205

[28] Cappella B, Baschieri P, Frediani C, Miccoli P, Ascoli C (1997) Force-distance curve by AFM. IEEE Eng Med Biol 16(2): 58–65. 10.1109/51.582177 9086373

[29] Laney DE, Garcia RA, Parson SM, Hansma HG (1997) Changes in the Elastic Properties of Cholinergic Synaptic Vesicles as Measured by Atomic Force Microscopy. Biophys J 72: 806–813. 9017205 10.1016/s0006-3495(97)78714-9 PMC1185603

[30] Radmacher M (2002) Measuring the elastic properties of living cells by the atomic force microscope. Methods Cell Biol 68: 67–90. 12053741 10.1016/s0091-679x(02)68005-7

[31] Liang X, Mao G, Simon Ng KY (2004) Probing small unilamellar EggPC vesicles on mica surface by atomic force microscopy. Colloids Surf B: Biointerfaces 34(1): 41–5. 15261089 10.1016/j.colsurfb.2003.10.017

[32] Dimitriadis EK, Horkay F, Maresca J, Kachar B, Chadwick RS (2002) Determination of Elastic Moduli of Thin Layers of Soft Material Using the Atomic Force Microscope. Biophys J 82(5): 2798–2810. 11964265 10.1016/S0006-3495(02)75620-8 PMC1302067

[33] Yin Z, Sadok A, Sailem H, McCarthy A, Xia X, et al (2013) A screen for morphological complexity identifies regulators of switch-like transitions between discrete cell shapes. Nat Cell Biol 15(7): 860–871. 23748611 10.1038/ncb2764 PMC3712499

[34] McBeath R, Pirone DM, Nelson CM, Bhadriraju K, Chen CS (2004) Cell shape, cytoskeletal tension, and RhoA regulate stem cell lineage commitment. Dev Cell 6(4): 483–495. 15068789 10.1016/s1534-5807(04)00075-9

[35] Cai X, Yang X, Cai J, Wu S, Chen Q (2010) Atomic force microscope-related study membrane-associated cytotoxicity in human pterygium fibroblasts induced by mitomycin C. J Phys Chem B 114(11): 3833–3839. 20196562 10.1021/jp910682q

[36] Girasole M, Pompeo G, Cricenti A, Congiu-Castellano A, Andreola F, et al (2007) Roughness of the plasma membrane as an independent morphological parameter to study RBCs: A quantitative atomic force microscopy investigation. Biochim Biophys Acta 1768(5): 1268–1276. 17320813 10.1016/j.bbamem.2007.01.014

[37] Teck Chwee Lim (2005) Single Cell Mechanics and its connections to Human Diseases. Asia-Pacific Biotech News 09: 674–675.

[38] Csaki C, Mobasheri A, Shakibaei M (2009) Synergistic chondroprotective effects of curcumin and resveratrol in human articular chondrocytes: inhibition of IL-1beta-induced NF-kappaB-mediated inflammation and apoptosis. Arthritis Res Ther 11(6): R165. 19889203 10.1186/ar2850 PMC3003513

[39] Liu FC, Hung LF, Wu WL, Chang DM, Huang CY, et al (2010) Chondroprotective effects and mechanisms of resveratrol in advanced glycation end products-stimulated chondrocytes. Arthritis Res Ther 12(5): R167. 20825639 10.1186/ar3127 PMC2990994

[40] Cai X, Yang X, Cai J, Wu S, Chen Q (2010) Atomic force microscope-related study membrane-associated cytotoxicity in human pterygium fibroblasts induced by mitomycin C. J Phys Chem B 114(11): 3833–3839. 20196562 10.1021/jp910682q

[41] Geiger B (2001) Cell biology: encounters in space. Science 294(5547): 1661–1663. 11721037 10.1126/science.1066919

[42] Hsieh CH, Lin YH, Lin S, Tsai-Wu JJ, Herbert Wu CH, et al (2008) Surface ultrastructure and mechanical property of human chondrocyte revealed by atomic force microscopy. Osteoarthritis Cartilage 16(4): 480–488. 17869545 10.1016/j.joca.2007.08.004

