# Supplementary figures and images for: Resveratrol Protects Chondrocytes from Apoptosis via Altering the Ultrastructural and Biomechanical Properties: An AFM Study
Source: PLoS One. 2014 Mar 14;9(3):e91611. doi: 10.1371/journal.pone.0091611 (PMC3954736; doi:10.1371/journal.pone.0091611)

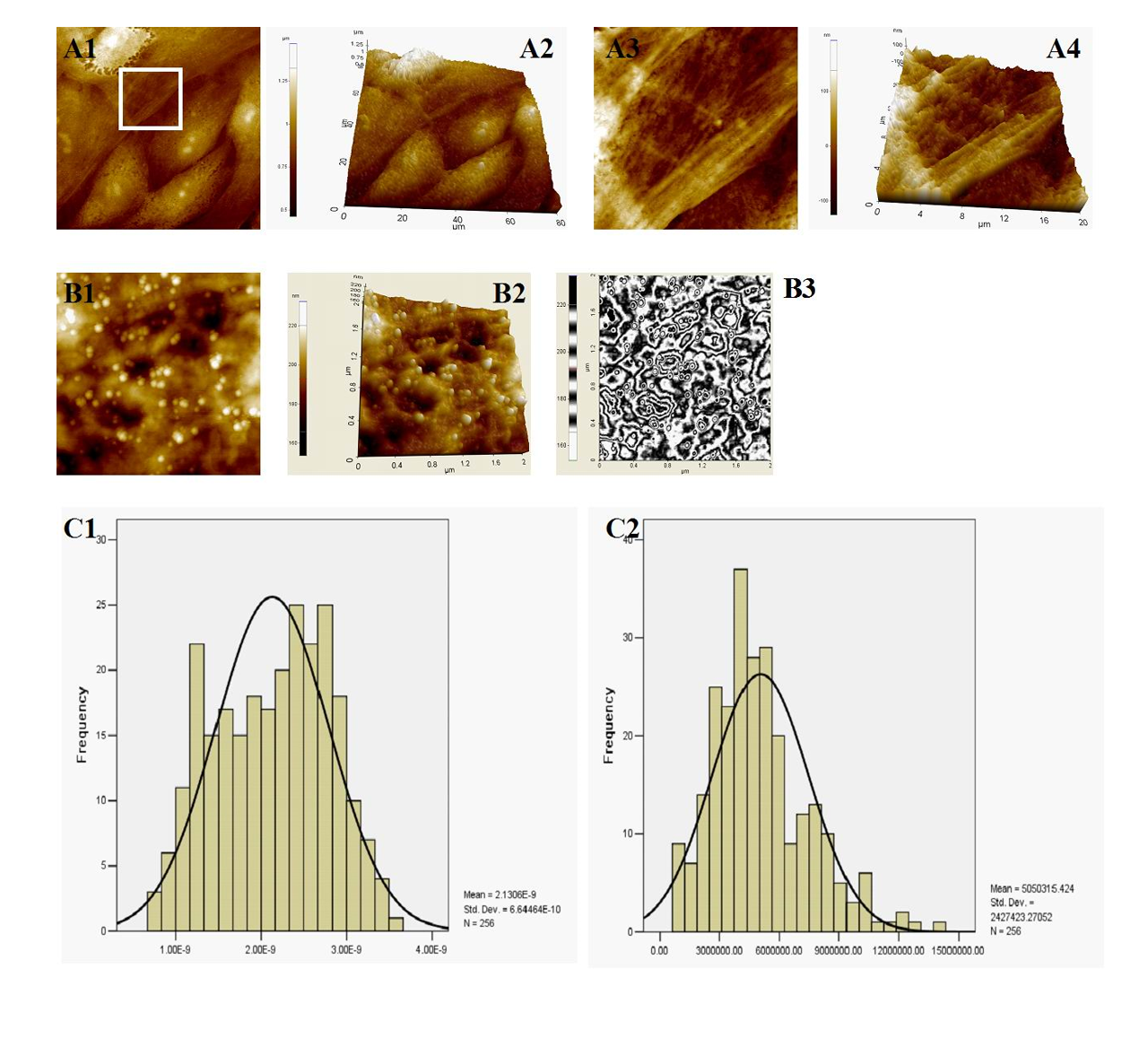

Supplement: Figure S1 — The morphological and nanomechanical properties of chondrocytes treated with 100 nM of RV for 24 h, which detected using AFM. A1–A2 Morphology of chondrocytes. A3–A4 the enlargement of white frame in A1. B1–B3 The ultrastructure of membrane on chondrocytes. C1,C2 The adhesion force and stiffness modulus of cellular membrane was 2.13±0.66 nN and 5.05±2.43 MPa. (TIF) [file pone.0091611.s001.tif]

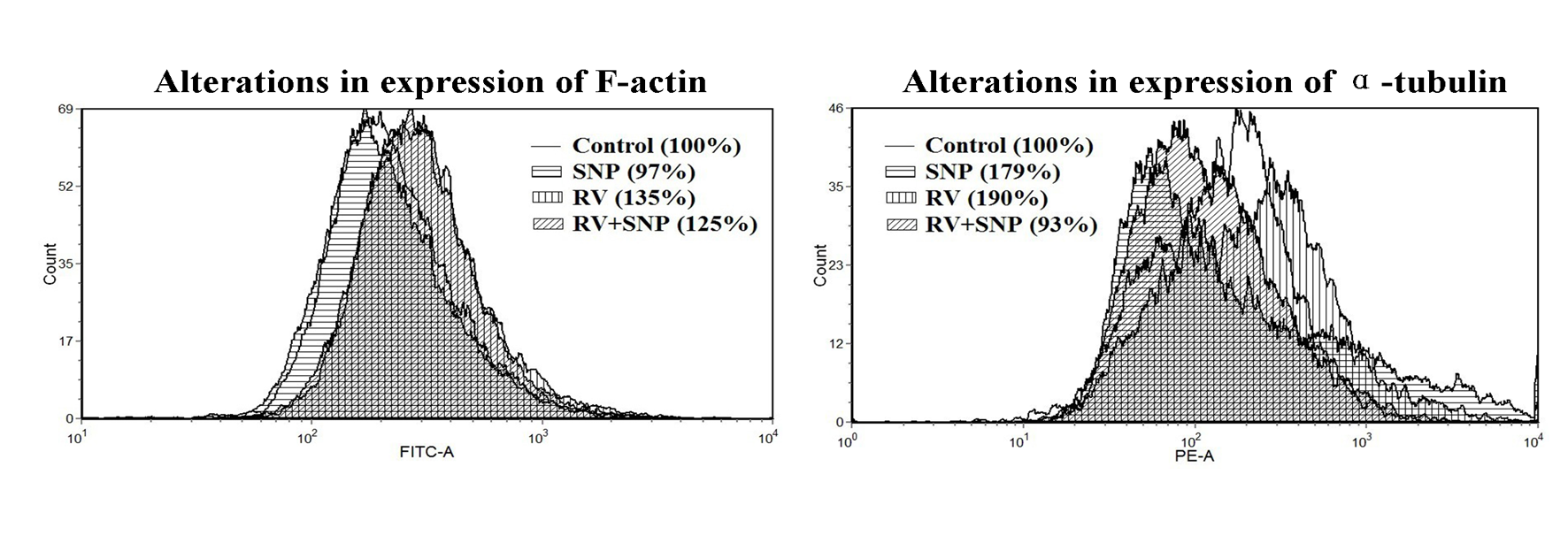

Supplement: Figure S2 — The alterations in expression of F-actin andα-tubulin in chondrocytes treated with SNP, RV and SNP+RV, respectively, which detected by fluorescence-based flow cytometry. The F-actins and microtubule were stained with FITC- phalloidine and Alexa Fluor 555-α-Tubulin antibody, respectively. (TIF) [file pone.0091611.s002.tif]
